# Response to physical activity of females with multiple sclerosis throughout the menstrual cycle: a protocol for a randomised crossover trial (EMMA Project)

**DOI:** 10.1136/bmjsem-2023-001797

**Published:** 2023-11-21

**Authors:** Jacobo Á Rubio-Arias, Domingo J Ramos-Campo, Nuria Romero-Parra, Luis Andreu-Caravaca, Alejandro Martínez-Rodríguez, Paula Esteban-García, Remedios López-Liria, Guadalupe Molina-Torres, Maria Isabel Ventura-Miranda, Ana Martos-Bonilla, Alberto Rando-Martín, Maria Carrasco-Poyatos, Fernando Alacid, María del Carmen Ferrer-Contreras, Rocio Cupeiro

**Affiliations:** 1 Health Research Centre, Humanidades-628 Research Group, Department of Education, University of Almeria, Almeria, Spain; 2 LFE Research Group, Department of Health and Human Performance, Faculty of Physical Activity and Sport Science (INEF), Universidad Politécnica de Madrid, Madrid, Spain; 3 Department of Physical Therapy, Occupational Therapy, Rehabilitation and Physical Medicine, Faculty of Health Sciences, Rey Juan Carlos University, Móstoles, Spain; 4 Sports Physiology Department, Faculty of Health Sciences, Universidad Católica de Murcia, Murcia, Spain; 5 Facultad de Deporte, Universidad Católica de Murcia, Murcia, Spain; 6 Department of Analytical Chemistry, Nutrition and Food Science, Alicante Institute for Health and Biomedical Research, Alicante, Spain; 7 Department of Physical Activity and Sports Sciences, Universidad de Castilla-La Mancha, Ciudad Real, Spain; 8 Department of Nursing, Physiotherapy and Medicine, University of Almería, Almería, Spain

**Keywords:** Fatigue, Quality of life, Muscle injury and inflammation

## Abstract

The relationship between multiple sclerosis (MS) and females is a crucial aspect in the development of the disease, with the ovarian hormonal cycle being a sensitive stage, especially in females with relapsing-remitting multiple sclerosis. The objectives of the study are to identify moderating variables that modify satisfaction with physical activity practice throughout the menstrual cycle (MC) in females in or out of their MC, during high-intensity interval training (HIIT) and strength training sessions and to compare the acute effects of different types of physical activity sessions in females with and without MS. This protocol is the methodology used in the EMMA Study, a randomised, single-blind crossover trial study conducted in females with MS who were matched 1:1, based on age, lifestyle factors and country of residence, with females without MS, to analyse the effect of physical activity practice on satisfaction, functionality, fatigue and inflammatory profile through their MC. Participants will visit the facilities approximately 10 times (4 preliminary familiarisation visits and 6 visits to carry out a physical activity session in each phase of the MC) for 3–4 months. A total sample of 30 females (15 females without MS and 15 with MS) is necessary for the study. The evaluation will comprise clinical, nutritional and psychological interviews, including different variables. It is hypothesised during the luteal phase, females with MS are expected to exhibit different acute responses to HIIT and strength training sessions as compared with females without the disease. Before starting the study, all participants will read and sign an informed consent form. Trial registration number: This research protocol is registered with ClinicalTrials.gov to ensure transparency and accessibility of study information (NCT06105463). The university’s ethics committee number for this study is UALBIO2022/048.

**WHAT IS ALREADY KNOWN ON THIS TOPIC**

- Multiple sclerosis (MS) predominantly affects females and exhibits a complex relationship with the menstrual cycle. Hormonal fluctuations during the menstrual cycle have been linked to changes in MS symptoms, but the acute responses to physical activity in different menstrual cycle phases are not well understood. Existing knowledge emphasises the importance of gender and hormonal factors in the clinical management of MS.

**WHAT THIS STUDY ADDS**

- This study, part of the EMMA Project, aims to investigate how satisfaction with physical activity varies throughout the menstrual cycle in females with and without MS. It also explores the acute effects of different physical activity sessions (high-intensity interval training and strength training). The study examines hormonal, functional and psychological aspects, shedding light on the interplay between MS, gender and the menstrual cycle, with implications for tailoring exercise regimens for individuals with MS.

**HOW THIS STUDY MIGHT AFFECT RESEARCH, PRACTICE OR POLICY**

- The EMMA Study can contribute to more personalised exercise programmes for females with MS by considering their menstrual cycle phase. The findings may provide insights into the role of hormonal fluctuations in symptom exacerbation and the effects of high-intensity interval training and strength training. This knowledge could enhance MS management strategies, emphasising gender-specific approaches and optimising outcomes for individuals with MS. The study’s results are expected to have significant implications for research, practice and policy, contributing to a better understanding of how the menstrual cycle affects the acute response to exercise in females with MS.

## Introduction

Multiple sclerosis (MS) is one of the most prevalent neurological diseases that affect young adults, typically occurring in individuals aged between 20 and 40 years old, with a higher incidence in females, in a ratio of approximately 2.3:3.5.^1^ The total prevalence is 33 cases per 100 000 people,^2^ affecting more than 2 million individuals worldwide.^3^ Interestingly, the global incidence and prevalence of MS are increasing, even in regions previously considered to be low risk.^4^ This chronic disease significantly affects individuals, leading to disability and impairment, making their personal, social and occupational lives very challenging.^5^

Although the aetiology of MS is unknown, it is believed to be a multifactorial disease resulting from the interaction of genetic, infectious and environmental factors (such as smoking, the ubiquitous γ-herpes virus, vitamin D deficit and exposure to sunlight).^6^ These factors, along with hormonal influences, contribute to the sexual differences observed in MS.^7^ Females experience more frequent relapses in relapsing-remitting MS (RRMS). They also develop more inflammatory lesions and have an earlier onset.^1^ Furthermore, the age of menarche is inversely associated with the risk of MS, and a lower relapse rate has been observed during pregnancy and menopause. In comparison, a higher relapse rate occurs after delivery.^8^ These findings highlight the importance of sex-specific approaches in the clinical management of MS, suggesting meaningful connections between gender, hormonal status and disease progression.

More specifically, menstruating females with MS during their premenstrual phase, and especially those with RRMS, could experience a worsening of symptoms due to exacerbation (new demyelinating plaques) and pseudoexacerbation (worsening of pre-existing deficits due to previous plaques).^7^ Additionally, body temperature fluctuates during the menstrual cycle (MC), increasing shortly after ovulation and remaining elevated throughout the luteal phase.^9^ This temperature increase can lead to the Uhthoff phenomenon, a temporary worsening or exacerbation of neurological symptoms due to heat.^7^ Furthermore, hormonal changes during the MC also moderate symptom fluctuations in females with MS.^9^

The long-term immunomodulatory effect of exercise is well-known. It has been shown that it can lead to a reduction in inflammation^10^ and the partial regulation of neuroimmune parameters in T cell behaviour.^11^ On the other hand, in healthy females, it has been observed that during physical activity (PA), hormonal fluctuations also lead to differences in performance and muscle damage. The perception of delayed-onset muscle soreness (DOMS) and loss of strength in response to PA is lower during the luteal phase when sex hormone concentrations are high. At the same time, during the early follicular phase (EFP), a greater perception of DOMS and a more significant loss of strength are observed.^12^ In this sense, the acute effects of exercise lead to an increase in T cell subsets 30 min after completing PA, followed by a decrease between 1 and 2 hours in healthy females. Training intensity modifies the immunomodulatory effect of T cell subpopulations and cytokines in healthy populations, generating a chronic response to immune system impairment.^13^ Therefore, PA could induce acute adverse events in females with MS during their reproductive years, especially during some phases of the MC, due to temperature changes and the acute inflammatory response to PA. However, to date, the acute response to exercise in females with MS and its relationship with hormonal fluctuations is still unclear.

Exercise has been proposed as a potential approach to improve MS symptoms, with aerobic exercise and strength training being the most commonly used and effective exercise modalities within the female population. Furthermore, it is worth noting that high-intensity interval training (HIIT) shows promise in enhancing fitness outcomes for individuals with MS.^14^ In this context, HIIT and strength training emerge as promising exercise strategies. However, achieving substantial benefits requires a minimum number of exercise sessions, underscoring the importance of adherence to the exercise programme when demonstrating positive effects.^15 16^ While the exact dropout and adherence rates in exercise programmes for MS remain unclear, several factors, such as age, gender distribution, disability level, exercise modality (HIIT vs strength training), intervention duration and specific exercises included, could potentially influence the dropout rate in physical exercise programmes.^17^ Hence, studying satisfaction with practising these exercises following a session is crucial. Understanding patients’ experiences and satisfaction levels can contribute to designing more effective and sustainable exercise programmes for those living with MS.

Even though age and gender are key factors that influence adherence and optimising outcomes, studies on MS and exercise often involve mixed groups comprised of both men and women without considering age or the biological phase. Additionally, there is a lack of exploration of acute responses during the different MC phases. Although some studies have examined the influence of MC on symptomatology, as well as on disease progression and rhythm,^7^ to our knowledge, no studies have specifically determined the acute response to PA in females based on the hormonal profile derived from the MC phases. Therefore, studies exclusively focused on females with MS are essential to understand better the potential relationship between MC, PA practice and MS.^18^ Furthermore, the relationship between MS and females is a crucial aspect in the development of the disease, with the ovarian hormonal cycle being a sensitive stage, especially in females with RRMS.

Thus, the objectives of the study are: (a) to identify moderating variables that modify satisfaction with HIIT and strength training sessions throughout the MC in females with and without MS, and (b) to compare the acute effects of different types of PA sessions (HIIT and strength training) throughout the MC, in females with and without MS.

The central hypotheses of this study are the following: first, during the luteal phase of the MC, females with MS are expected to show a stronger association between inflammation and perception (fatigue and energy) and hormonal levels, as compared with females without the disease. Furthermore, HIIT and strength training sessions are expected to impact satisfaction with PA practice in both populations throughout the MC. During the luteal phase of the MC, females with MS are expected to exhibit different acute responses to HIIT and strength training sessions as compared with females without the disease.

## Material and methods

### Design

**Figure 1:**
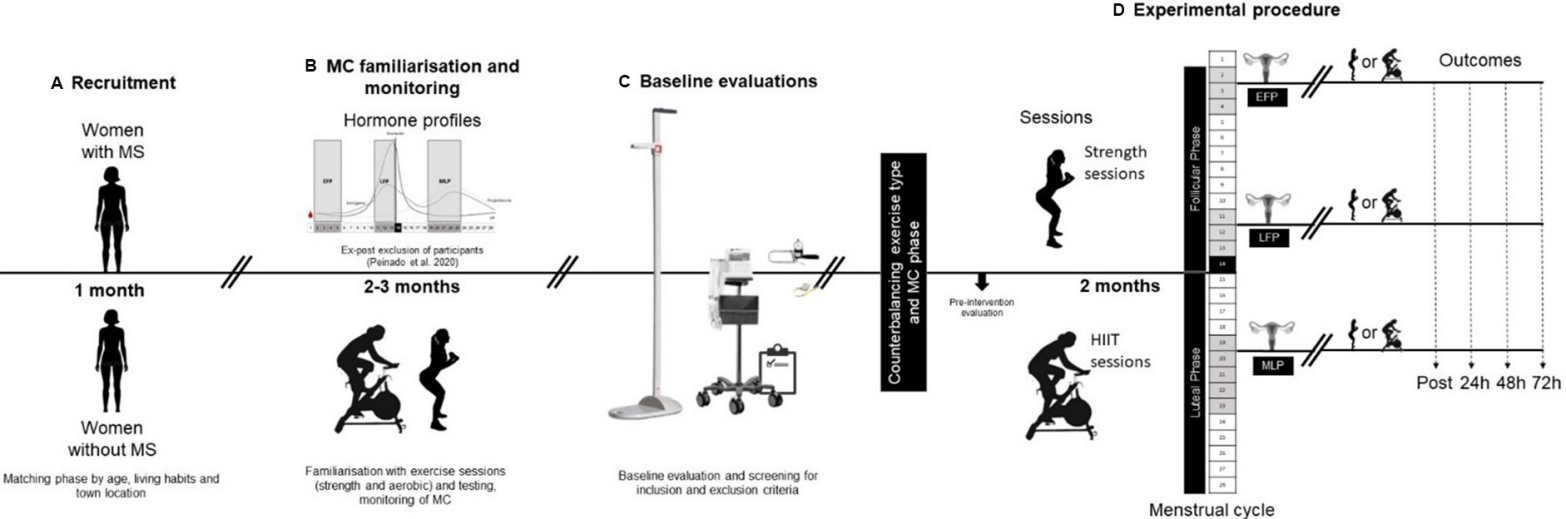
Experimental design. EFP, early follicular phase; HIIT, high-intensity interval training; LFP, late follicular phase; MC, menstrual cycle; MLP, mid-luteal phase; MS, multiple sclerosis; post, immediately after exercise session.

The present protocol describes the methodology of the EMMA Study, a randomised, single-blind crossover trial conducted on females with MS, matched 1:1 based on age, lifestyle factors (smoking, PA) and country of residence, with females without MS, to analyse the effect of PA practice (HIIT vs strength training) throughout the MC. To achieve this, the Consolidated Standards of Reporting Trials recommendations and protocols^19^ and previous studies that analysed the impact of the MC in females without MS^20^ will be followed. Participants will visit the facilities approximately 10 times (4 preliminary familiarisation visits and 6 visits to carry out HIIT and strength training session in each phase of the MC) over 3–4 months (figure 1).

### Participants

Females diagnosed with RRMS according to McDonald’s criteria^21^ and certified by their neurologist will be invited to participate in the study voluntarily. Recruitment will be carried out in collaboration with Regional Multiple Sclerosis Associations and hospitals in the provinces of Almería, Alicante, Jaén, Toledo and Murcia. The established inclusion and exclusion criteria will be analysed for all females who voluntarily express their interest in participating.

An a priori sample size estimate was performed using G*Power V.3.1 software (Heinrich-Heine-University, Düsseldorf, Germany) to estimate the number of females required for the study. An a priori sample size estimation was performed, setting the significance level at α=0.05, the power at β=0.95, a large effect size of f(v)=0.80 and a sphericity correction of 0.2 (1/repetitions−1). Additionally, a minimum dropout rate of 25% was considered following Hopkins *et al*’s recommendations.^22^ Since the study aims to analyse the effect of hormonal fluctuations, a previous study that analysed hormonal differences in females with regular cycles was used.^23^ A sample of 60 females (30 females without MS and 30 with MS) is necessary for the study. In the current study, the sample size is larger than in previous clinical trials that analysed the acute effects of PA on MS.^24^ Nevertheless, several studies have employed sample sizes ranging from 15 to 20 individuals with sclerosis and 15–20 without sclerosis. Therefore, the study will consider a minimum of 15 per control group.^25 26^

The inclusion criteria will be as follows: (a) women aged between 18 and 40 years old; (b) females with an MC length of ≥21 days and ≤35 days of natural menstruation; (c) absence of iron deficiency anaemia (serum ferritin >20 µg/L, haemoglobin >115 µg/L and transferrin saturation >16%); (d) being in a stable phase of the disease; and (e) to ambulate autonomously for more than 100 m. Participants will be excluded if they have: (a) a score <2 or >6 on the Expanded Disability Status Scale; (b) experienced a relapse in the 12 months before enrolment; (c) received corticosteroid treatment in the previous 2 months; (d) participated in a structured exercise programme in the past 6 months; (e) secondary amenorrhoea (absence of ≥3 consecutive periods despite not being pregnant and having previous menstruation); (f) used or currently using hormonal contraceptives for 3 months before recruitment; (g) reported musculoskeletal or neurological injuries not associated with MS, recent surgical interventions or pregnancies in the previous year; (h) diseases unrelated to MS. Furthermore, exercise and dietary restrictions and recommendations will be established 24 hours before the sessions, on the intervention day, and 24, 48 and 72 hours after, following the recommendations outlined in Peinado *et al*.^20^ Participants will be instructed not to modify their lifestyle during their participation in the project.

In addition to these inclusion and exclusion criteria, participants will be required to provide information, following the recommendations of Elliott-Sale *et al*,^27^ pertaining to the number of pregnancies (single or multiple pregnancies); age at menarche and any complications; previous MC irregularities; contraceptive use in the 12 months before the study, specifying the type (implants, injections, intrauterine devices/hormonal intrauterine systems, vaginal rings, transdermal patches) and formulation; the number of pregnancies resulting in gestational age of 24 weeks or more, regardless of whether the child was born alive or stillborn. Additionally, data on habitual PA (International Physical Activity Questionnaire), daily caffeine intake, dietary intake and nutritional supplements, alcohol consumption and smoking will be collected. Participants will also be asked to indicate the date of their MS diagnosis and the number of relapses.

Once all females with MS have been recruited, the enrolment of females without MS will begin, using a 1:1 matching methodology based on age, geographical area, lifestyle factors, and inclusion and exclusion criteria related to the MC. Before starting the study, all participants will read and sign an informed consent form. Furthermore, the project will adhere to the ethical principles of medical research involving human subjects described in the Declaration of Helsinki of the World Medical Association. This project and its procedures have also been registered in the US National Library of Medicine’s ClinicalTrials.gov database (NCT06105463).

### Procedure

The methodological guidelines established by Elliott-Sale *et al*^27^ for research in sports and exercise sciences with female participants will be followed to conduct the study. In addition, the recommendations proposed by Schmalenberger *et al*^28^ and Janse de Jonge *et al* for studying the MC will also be considered.^29^

#### Familiarisation, MC monitoring and determination of MC phases

**Figure 2:**
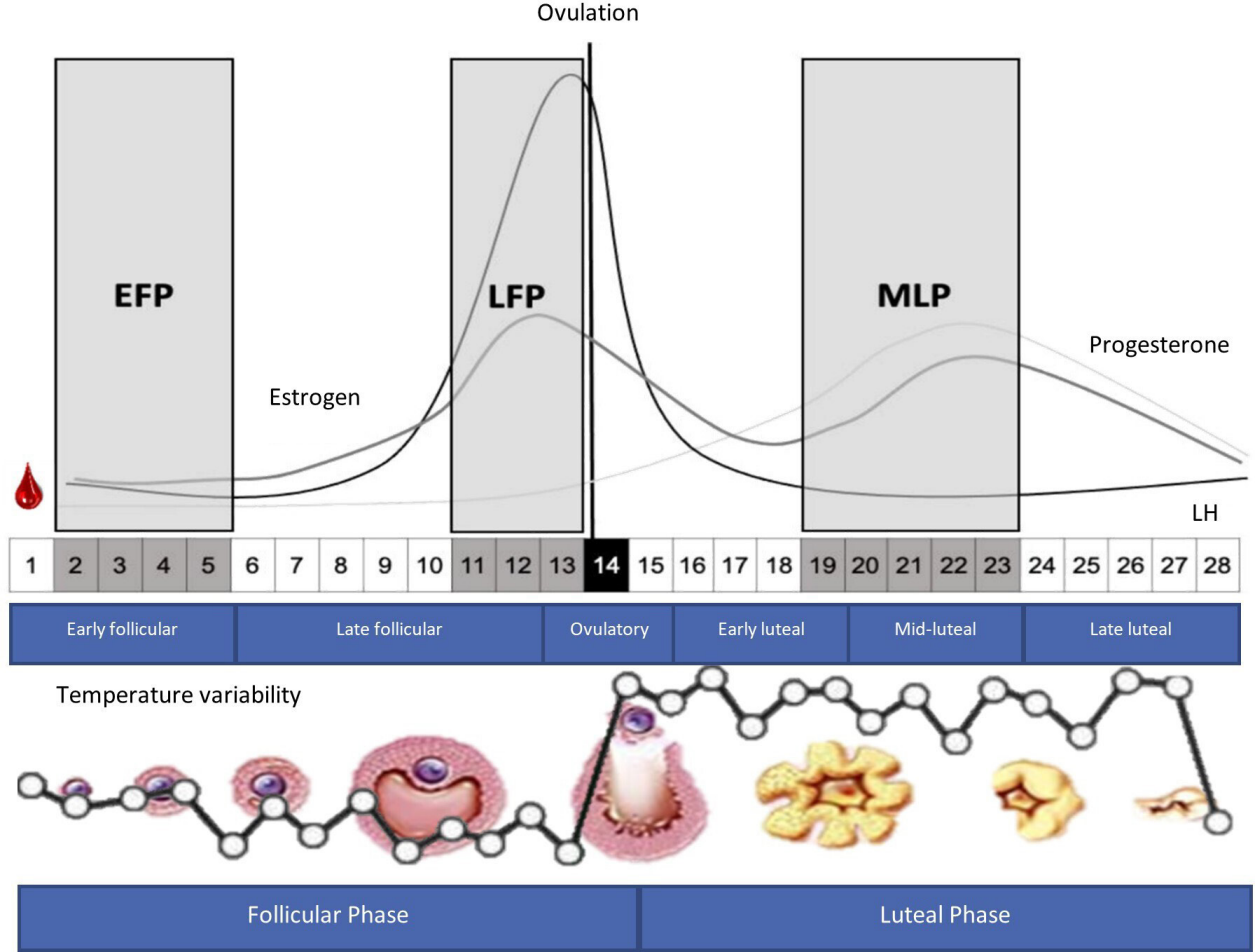
A depiction of a 28-day menstrual cycle with ovulation on day 14, showing the typical fluctuations in ovarian hormones, such as oestrogen, progesterone and luteinising hormone (LH), as well as the changes in temperature, during the follicular and luteal phases. Modified from Peinado *et al*^20^ and Schmalenberger *et al.*^28^ Timing of sessions for an average duration cycle (28 days): from the first day of bleeding, the tests will be performed between the first and fifth day (EFP=low levels of sex hormones), between days 11 and 13 (LH peak or the same day; LFP=low levels of progesterone but high oestrogen levels), and between days 19 and 23 (after ovulation; MLP=elevated levels of both progesterone and oestrogen). EFP, early follicular phase; LFP, late follicular phase; MLP, mid-luteal phase.

For this study, three out of the widely considered four phases of the MC will be selected: phase 1 (EFP), when oestrogen and progesterone are at their lowest blood concentrations; phase 2 (late follicular phase: LFP), when the highest oestrogen concentration occurs while progesterone remains low; and phase 4 (mid-luteal phase: MLP), the highest concentration of progesterone and a high concentration of oestrogen occur (figure 2). These phases will be identified following the protocols proposed by Peinado *et al*^20^ and Janse de Jonge *et al*.^29^

During the 2–3 months before the intervention, familiarisation with the tests and PA sessions will be conducted. This familiarisation period will start with MC monitoring, allowing the appointment setting for/in each MC phase with little margin of error. For the monitoring of the MC and the setting of the appointments, the experimental considerations described below will be followed^27 29^:

- Monitoring will be carried out starting 3 months before starting the intervention. Participants will be requested to provide information regarding the starting date of their six previous menstruations to estimate their MC duration. Then, during the two to three subsequent MCs, period starting dates will be collected concurrently with the familiarisation stage.
- Luteinising hormone (LH) peak detection urine kits will detect LH surge, ovulation, and, retrospectively, the LFP. This test will allow us to confirm that the MCs included in the study are ovulatory, as anovulatory cycles can occur even in normal cycles with regular menstruation.^29^
- In addition, serum hormone concentrations (see Reproductive hormonal profile subsection) will be measured for each training session retrospectively since this is a direct method and is considered the gold standard for research purposes.

Therefore, a three-step method will be used for MC phase confirmation, combining the calendar method, qualitative determination of LH peak in urine and analysis of serum hormones, following the protocols described by Peinado *et al*^20^ and Janse de Jonge *et al*.^29^ After the familiarisation period, a posteriori exclusion of participants and/or cycles who/which do not fulfil the levels of oestrogen and/or progesterone concentrations expected for each phase will be applied.

Additionally, familiarisation sessions will be conducted, employing the exercises used in the HIIT and strength training sessions, and these sessions will also be used to estimate the 1-RM (repetition maximum) and maximal oxygen consumption (VO_2_max) of each participant.

#### Randomisation

In this crossover study, the evaluation order will be counterbalanced according to the MC phases (EFP, LFP and MLP) using codes 1–3. Participants and their counterparts will undergo evaluations in different sequences: 1–2–3; 2–3–1; 3–1–2; 2–1–3; and 1–3–2.^20^ Additionally, the type of exercise (HIIT: 1 or strength training: 2) they will perform in each phase will be randomised. This randomisation approach will ensure an equitable distribution of the effects of the MC and the types of exercise among participants, contributing to obtaining more robust and reliable results for the analysis. The ‘OxMaR’ software will be used for the participant randomisation process.^30^

#### Experimental procedures

All sessions will be conducted at the same time of day to avoid different responses to changes in circadian rhythm, and the temperature (21–22°C) and humidity (45–60%) of the evaluation areas will be controlled. In the first visit, fasting (4 hours) and resting without exercising in the previous 24 hours will be required. First, participants will be informed about the study’s objectives, sign the informed consent forms, complete the health and lifestyle questionnaires, and the inclusion and exclusion criteria will be assessed. Subsequently, descriptive data will be collected, and body composition and anthropometric tests will be performed. Finally, blood samples will be collected. Second and third visits will be performed to familiarise the participants with the maximal ramp test on a cycle ergometer and 1-RM exercise to individualise the physical exercise sessions and to familiarise them with the different tests. Subsequent visits will be scheduled according to the timing of the MC phases estimated in the monitoring months. A pre-intervention, immediate post-intervention, 24, 48 and 72-hour post-intervention assessment will be performed at each MC phase (hormone profile). A HITT and strength training session will be performed between the pre-assignment and post-assignment. Table 1 shows the activities to be performed in each MC. This procedure will be performed twice (HIIT and strength training sessions). All outcomes will be assessed by study staff blinded to group assignments as far as possible.

**Table 1:** Study activities

Outcomes	Baseline measurements in each cycle (1 time/cycle)	Baseline measurements in each phase of the MC (3 times/cycle)	Pre-sessions	Post-sessions	24 hours post-sessions	48 hours post-sessions	72 hours post-sessions
Primary outcomes							
Satisfaction			x	x	x	x	x
Visual Analogue Scale for Fatigue (VAS-F)			x	x	x	x	x
Secondary outcomes							
Abdominal obesity and anthropometric variables	x						
Dietary and nutritional monitoring	x						
Monitoring of PA levels	x						
Walking endurance (2-MWT)	x						
Physical self-perception		x					
Catastrophizing Pain Scale		x					
Modified Fatigue Impact Scale		x					
Multiple Sclerosis Quality of Life-54		x					
State anxiety and trait anxiety		x					
Body temperature		x					
Bioimpedance analysis		x					
Reproductive hormonal profile			x				
Inflammatory profiles			x	x*			
Cognitive profiles			x	x*			
Neuromuscular strength, voluntary activation and contractile properties: RFD, MVIC, CAR, MULS			x	x	x	x	x
Functional assessment: spasticity, gait speed, balance, STS, TUG			x	x	x	x	x
Rating of Perceived Exertion			x	x			
Pain: VAS, algometer			x	x	x	x	x
Muscle oxygen saturation			During sessions				
Lactate			x	x†			
VO_2_max			During sessions				
Heart rate variability (HRV)			During sessions				
Quality of sleep: Karolinska Sleep Diary Questionnaire, actigraphy-based sleep quality, HRV					x	x	x

*It will be assessed immediately, at 30 min and 2 hours after the end of the session.

†It will be assessed immediately, at 3 min and 30 min after test.

CAR, central activation ratio; MC, menstrual cycle; MULS, maximal upper limb strength; MVIC, maximal voluntary isometric strength of the lower limbs; 2-MWT, 2-Minute Walk Test; PA, physical activity; RFD, rate force development; STS, sit-to-stand; TUG, timed up and go test; VO_2_max, maximal oxygen consumption.

### Exercise training sessions

The characteristics of each HIIT and strength training session that participants will perform have been designed according to the recommendations provided by guidelines^31^ and results reported in recent meta-analyses published by Andreu-Caravaca *et al*^15 16^ and Campbell *et al*,^14^ to optimise the long-term results of physical exercise programmes.

#### HIIT session protocol

The proposed protocol for the HIIT session is based on previous studies conducted with this population on a cycle ergometer, with the following distribution: 5 min of warm-up at 50% of peak power+20 min of HIIT consisting of 30 s at 90% of peak power, followed by 30 s of passive rest+5 min of cool-down at 35–40% of peak power. Participants will be asked to maintain a cadence between 50 and 80 revolutions per minute (rpm); if participants do not achieve 50 rpm, they will be encouraged to cycle as fast as they can (but not less than 40 rpm). Peak power will be previously determined through an incremental cycle ergometer test using the cycle ergometer (Ergoselect 200, Ergoline, Germany), which will include 5 min of warm-up with unloaded cycling followed by a ramp incremental test with 25 W increments every 2 min until exhaustion. The test will be terminated when the participants reach volitional exhaustion or the cadence drops by 10 rpm.^25 32^ During the test and the sessions, the heart rate will be measured by an H10 heart rate monitor (Polar Electro, Kempele, Finland), and the cardiorespiratory response will be analysed using a gas analyser VO2 Master Pro (VO2 Master Health Sensors, Vernon, British Columbia, Canada). Peak power will also be established during each of the MC phases. Peak power will serve as a baseline at each of the hormone phases.

#### Strength training session protocol

Participants will perform a standardised warm-up protocol (5 min on a stationary bicycle, mobility of the lower extremities and 5 repetitions at 40% 1-RM on each machine) before each strength training session. Subsequently, participants will perform four lower limb exercises, including bilateral leg press, unilateral leg extension, unilateral hip extension and bilateral seated calf raise on conventional weight machines (Fittech, Viseu, Portugal). The session’s intensity will be 70–75% of 1-RM, performing a volume of 4 sets of 10 repetitions, leaving 1–2 repetitions in reserve (RIRs) and 120-second rest between sets. The 1-RM will be calculated for each exercise in the familiarisation phase.

The 1-RM load will be estimated using the following protocol: 1 set of 10 repetitions at 50% of the estimated 1-RM, 1 set of 5 repetitions at 75% of the estimated 1-RM, finishing with 1 set of 1 repetition at 100% of 1-RM. A 5-minute rest period will be allowed between sets. If a participant can complete more than one repetition in the last set, the 1-RM will be estimated based on recommendations published in the literature. Supervisors will instruct participants to lower the weight in a controlled manner, pause briefly at the end of the movement and then contract the muscle as fast as possible (concentric phase) to maximise the neural component. In the training session, the load will be increased by 2–5% when the participants can achieve two more repetitions than the predetermined ones, always with two RIRs.

During the session, the supervisors will fill out a control sheet where the weight lifted in each exercise, the completed repetitions and sets, and RIR will be recorded. All sessions will be supervised by the same certified trainer, specialised in strength training. The 1-RM will be established during the control phases of the MC, and a baseline will be set at different points in the hormonal phase for the study.

### Outcome measures

All outcomes will be conducted and interpreted by study personnel who are blinded to group allocation (table 1).

#### Primary outcomes

##### Satisfaction

Satisfaction with PA will be assessed using an eight-item scale adapted from previous studies.^33 34^ The questionnaire begins with the statement ‘When I am doing PA’, followed by the items ‘I am satisfied with the results of/I am satisfied with/I enjoy/I feel good when I have done/I notice positive results if I have done PA’, ‘PA has many advantages’ and ‘I find PA nice/difficult’. Responses to this single-item question will be measured on a 5-point Likert scale, ranging from 1 (very dissatisfied) to 5 (very satisfied). Satisfaction will be assessed before, immediately following the training session, and at 24, 48 and 72 hours.

##### Visual Analogue Scale for Fatigue

Visual Analogue Scale for Fatigue (VAS-F): to measure fatigue, the VAS-F will be used. The VAS-F will be assessed before, immediately following the training session, and at 24, 48 and 72 hours. This scale, developed by Lee *et al*,^35^ is subdivided into two subscales: fatigue and energy. The VAS-F presents a horizontal line measuring 100 mm in length, with the term ‘none’ at one end and ‘very severe’ at the opposite end. Participants must mark the point on the line corresponding to their perception of fatigue severity between these two endpoints. The fatigue subscale is organised, from the most positive to negative items. Conversely, the energy subscale ranges from the most negative to the most positive items. A high score on the VAS-F indicates a low score on the energy subscale and a high severity level on the fatigue subscale.^36^ This scale is widely used in the general population and clinical patients due to its brevity, ease of use and comprehensibility. The fatigue subscale of the VAS-F shows a Cronbach’s α of internal consistency of 0.90. The Cronbach’s α for the energy subscale is 0.74. Participants will be instructed to consider their overall level of fatigue when responding to the scale rather than a specific moment.

#### Secondary outcomes

Secondary outcomes will be assessed at various time points, with specific protocols outlined in online supplemental material.

10.1136/bmjsem-2023-001797.supp1 Supplementary data

##### Baseline outcomes measured in each MC (control outcomes)

Baseline assessments will be conducted in each MC, including evaluations of abdominal obesity and anthropometric variables, dietary and nutritional monitoring, monitoring of PA levels and walking endurance.

##### Measurements in each phase of the MC

Throughout different phases of the MC, various measurements will be carried out. These assessments include the evaluation of physical self-perception, assessment using the Catastrophizing Pain Scale, analysis of the Modified Fatigue Impact Scale, examination of the Multiple Sclerosis Quality of Life-54, evaluation of state anxiety and trait anxiety, monitoring of body temperature and bioimpedance analysis.

##### Assessments before and after each physical exercise training session

Before and after each physical exercise training session, the following evaluations will take place: measurement of reproductive hormones, including 17β-oestradiol, progesterone, prolactin, LH, follicle-stimulating hormone, thyroid-stimulating hormone and testosterone; analysis of inflammatory markers, such as interleukin (IL)-6 and IL-10; assessment of cognitive function, including brain-derived neurotrophic factor; examination of the blood profile; evaluation of neuromuscular strength, voluntary activation and contractile properties, including rate force development, maximal voluntary isometric strength of lower limbs, muscle contractile function and central activation ratio; and analysis of maximal upper limb strength. Functional assessment includes evaluating spasticity, gait speed, balance, the sit-to-stand test, the timed up and go test, pain levels and the rating of perceived exertion.

##### Evaluations during intervention sessions (per session)

During each intervention session, the following evaluations will be conducted: measurement of muscle oxygen saturation, analysis of lactate levels, assessment of VO_2_max and monitoring of heart rate variability (HRV).

##### Assessing residual effects of exercise training sessions

Sleep quality will be evaluated over the three nights following each intervention to assess the lingering effects of exercise training sessions. This assessment will use the Subjective Sleep Quality Questionnaire, sleep quality measured by actigraphy and HRV.

## Data Availability

No data are available. All data relevant to the study are included in the article or uploaded as online supplemental information.
