# Supplementary material for: Response to physical activity of females with multiple sclerosis throughout the menstrual cycle: a protocol for a randomised crossover trial (EMMA Project)
Source: BMJ Open Sport Exerc Med. 2023 Nov 21;9(4):e001797. doi: 10.1136/bmjsem-2023-001797 (PMC10668283; doi:10.1136/bmjsem-2023-001797)
Supplement: Supplementary data [file bmjsem-2023-001797supp001.pdf]

Supplementary Material 1. Description of the procedures for the secondary variables

1. Secondary outcomes

1.1. Baseline outcomes measured on each menstrual cycle (control outcomes)

Abdominal Obesity and Anthropometric Variables

Body mass, stature, relaxed arm, mid-thigh, calf, waist and hip girths, and three skinfolds (triceps, front thigh and medial calf) will be measured, following the protocol established by The International Society for the Advancement of Kinanthropometry (ISAK), by an ISAK level 1 certified anthropometrist [1]. The following will be calculated with the data obtained: BMI (Weight (kg)/ (Height (m))2, waist-to-hip ratio (WHR) and waist-to-height ratio (WHtR). Abdominal obesity will be identified based on WHtR when it is over 0.5 [2.]

In addition, relative fat mass (RFM), will also be estimated using the following equation: RFM (%) = 64 - (20 · height/waist girth) + 12 [3]. Additionally, Skeletal muscle mass (SMM) will be determined by applying [PEG2] the equation "SMM (kg) = 0.566 * FFM, followed by the calculation of the skeletal muscle mass index (SMMI) using the formula "SMMI = SMM (kg) / height squared (m2) [4]. Finally, the prevalence of sarcopenia among the participants will also be calculated following the criteria of the European Working Group on Sarcopenia in Older People (EWGSOP) [5].

Dietary and nutritional monitoring

To establish both an initial baseline and a monthly evaluative measurement of dietary practices and adherence, the aforementioned 'Dietary and Nutritional Monitoring Protocol' will be administered at two critical junctures of our study. Initially, at the onset of the research protocol, this dietary assessment will be undertaken to capture the existing eating patterns, providing a pre-intervention description of participants' diets. This data will be instrumental in identifying any subsequent changes or trends potentially attributable to the intervention. At the conclusion of each month throughout the study, the same dietary assessment will be administered again, ensuring monthly post-intervention records of dietary habits. The comparison between these monthly datasets will provide a comprehensive perspective on shifts in dietary patterns and adherence throughout the study.

Following the outlined schedule for dietary assessment, participants will be guided through the core elements of the "Dietary and Nutritional Monitoring Protocol." A significant component of this monitoring process will be the detailed questionnaires targeting the Mediterranean diet's principles (PREDIMED Questionnaire [6,7];

Mediterranean Diet Adherence Screener (MEDAS) [8,9]). This structured inquiry is tailored to distill the intricacies of each participant's adherence to the diet, shedding light on their individual dietary habits.

Subsequently, over a span of one week, participants will be instructed to maintain an exhaustive food diary. They will be asked to provide entries detailing not only the foods and beverages consumed, but also their precise quantities. The added requirement of a photographic record of each meal and snack is designed to further confirm portion sizes and the nature of foods consumed. This visual reinforcement seeks to add another dimension of accuracy to the data.

Weighing of food items will be highlighted as an essential practice. Through clear guidelines, participants will be made aware of the nuances of weighing foods, especially the disparities between the weights of raw versus cooked items. Anticipating potential challenges, we've also prepared an auxiliary guide on household measures, serving as an alternative when traditional weighing methods prove to be impractical.

Ensuring clarity and addressing uncertainties, participants will have the advantage of being in direct touch with a registered dietitian-nutritionist. This continuous professional support is aimed at guiding participants through the intricacies of accurate food recording. Ultimately, the dietitian-nutritionist will bear the responsibility of analysing the amassed data, validating its credibility.

Monitoring of Physical Activity levels

The International PA Questionnaire (IPAQ) [10,11], will be administered once in each MC as a control variable. The telephone-based short version, consisting of 7 items, will be utilized. This version provides information about the time spent performing moderate and vigorous-intensity activities, time dedicated to walking, and time spent sitting during a workday. It is designed to be used with young people and adults aged 15-69 years old. PA will be recorded in METs (Metabolic Equivalent of Task or METs Units) using the following equation, only at the beginning and end of the experimental design (control measure):

$$\begin{aligned}\text{MET} - \text{minutes/week} &= \text{walking } (3'3 \text{ MET} \times \text{minutes} \times \text{days per week}) \\ &+ \text{moderate PA } (4 \text{ MET} \times \text{minutes} \times \text{days per week}) \\ &+ \text{high PA } (8 \text{ MET} \times \text{minutes} \times \text{days per week}).\end{aligned}$$

In addition, the participants' PA will be assessed with WGT3X-BT triaxial accelerometers (Cambridge Neurotechnology, Cambridge, UK) for 7 days of the experimental period to verify PA levels.

Walking endurance

Participants will undergo the 2-minute walk test (2-MWT), during which they will walk at their self-selected preferred speed to assess walking endurance. The testing track will be rectangular, with corners marked by cones. Participants will be allowed to rest during the test if needed, but the time will not pause during these resting periods. The total distance covered (in meters) will be recorded. An investigator will be present during the test to accompany the participants, although no conversations will take place [12].

Measurements in each phase of menstrual cycle

Physical self-perception.

The evaluation of physical self-perception [13] will comprise six subscales aimed at gauging self-perception in various dimensions, including sports competence, physical condition, attractive body, physical strength, general physical self-perception, and general self-perception. The participants' responses will be organized using a 5-point Likert scale, with each subscale score having the potential to obtain 6 to 36 points. A higher score will indicate a positive physical self-perception.

Catastrophizing Pain Scale

The Catastrophizing Pain Scale [14] will be used to assess feelings of catastrophizing related to pain (such as painful experiences). Subscale scores encompassing rumination and helplessness will be examined. Each of the 13 questions will be rated on a 5-point Likert scale, with the endpoints ranging from <0> "not at all" to <4> "all the time." A lower score will indicate minimal or no pain catastrophizing.

Modified Fatigue Impact Scale (MFIS)

The perception of fatigue will be measured using the MFIS. This scale is a 21-item multidimensional questionnaire that assesses the physical, cognitive, and psychosocial effects of fatigue on a five-point ordinal scale (with a maximum total score of 84) [15].

Multiple Sclerosis Quality of Life-54 (MSQoL-54)

Participants will filled out the Multiple Sclerosis Quality of Life-54 (MSQoL-54) questionnaire. The MSQoL-54 is a structured, self-report questionnaire comprising 14 sub-scales: physical function, physical role limitations, emotional role limitations, pain, emotional well-being, energy, health perceptions, social function, cognitive function, health distress, sexual function, satisfaction with sexual function, change in health, and

overall quality of life. Two summary scores, the physical health composite summary and the mental health composite summary, can be derived from the MSQoL-54 questionnaire. Elevated scores in each subscale or summary score will indicate an improved quality of life.

State anxiety and trait anxiety (STAI)

The STAI will be used to measure state and trait anxiety. This questionnaire assesses and discriminates the temporary psychological state in a given situation (state anxiety; 20 items), as well as the more stable character trait of attitudes and temperaments (trait anxiety; 20 items) [16]. This questionnaire is especially useful for the diagnosis of anxiety problems in non-psychiatric patients. It is based on a 4-point Likert scale, with scores in each subscale ranging from 10 to 40 points. A higher score in each of the subscales reflects a higher state or trait anxiety, respectively.

Body temperature

Body temperature will be measured through thermal images. To capture thermal images, a FLIR C5 thermographic camera (FLIR, Estonia) will be used, equipped with an infrared resolution of 19200 pixels and a thermal temperature range of -10 to 50ºC (Thermal sensitivity/NETD <70 mK). The calibration of the camera will be performed according to the manufacturer's recommendations, as well as recommendations from previous studies [17,18]. The camera will be connected at least 30 min prior to all evaluations to allow its thermal sensor to stabilise.

Three measurements shall be performed following the Glamorgan protocol [18], designed to provide accurate measurements of body thermography. This procedure will be repeated at each phase of the MC. Image collection will be performed in a laboratory with standardised conditions, and monitored according to the specific recommendations for this type of evaluation [17,19]. The procedure will start with a baseline thermography, followed by a thermography after mechanical stress (physical exercise), and finally a thermography after thermal stress. To ensure the thermal stability of the participants, they will be allowed a 10-minute acclimatisation period to the ambient temperature of the laboratory [20]. During the acclimatisation period, they will be asked to answer a questionnaire to ascertain the existence of any possible influencing factors affecting the results. The thermal imaging camera will be positioned perpendicular to the ground at a specific distance (2 meters), which will allow the subject to fit completely in the camera image, thus ensuring that all ROIs (Regions of Interest) can be satisfactorily assessed. The images will be analysed with the Irbis®3 software. The ROIs will be: trunk, hip and lower limbs following indications from previous studies.

Bioimpedance analysis

Body composition will be measured by bioelectric impedance analysis (BIA) using a portable device (InbodyS10® system, InBody Corp, Seoul, Korea), with an applied current of 100 μA (1 KHz) and 500 μA (5, 5, 50, 250, 500, and 1000 kHz), and a 100-240 VAC power supply, 50 / 60 Hz, 1.2 A, in five segments of the body (right and left arms, right and left legs, and trunk). The parameters obtained in the measurement will include the following: weight, lean muscle mass (muscle mass), skeletal muscle index (skeletal muscle mass/height [m^2]), fat-free mass, skeletal muscle mass, body fat percentage, extracellular water/total body water ratio (ECW/TBW), basal metabolic rate, and phase angle arc tangent of (Xc/R) × 180°/π), which will be automatically calculated by the device.

1.2. Before and after each physical exercise training session

Reproductive hormones, inflammatory and cognitive function, blood profile

Methodological considerations for performing blood collections: as a critical component of our upcoming research, we've established a 4-hour fasting protocol. This method ensures standardization across participants and aims to create a consistent metabolic baseline, allowing for more accurate and reliable study outcomes. In the upcoming study, participants will be informed about the significance and objectives of the fasting protocol. They will receive recommendations for a standard meal to consume before the fasting period begins, ensuring a controlled intake of calories and macronutrients. Furthermore, participants will be advised to avoid strenuous activities or exercises in the 24 hours leading up to the fasting period. For dinner, the total caloric intake will be set at 450 kcal. Carbohydrates make up 50% of this, which is equivalent to 225 kcal or 56 grams. Proteins contribute 25%, corresponding to 113 kcal or 28 grams. Similarly, fats also account for 25% of the meal's energy, amounting to 113 kcal or 12 grams.

Participants will also receive instructions regarding medications during this period. Some may need to take their medication with food, so consultation with a medical professional will be necessary to offer guidance. The use of non-essential supplements will be discouraged during the fasting period unless otherwise instructed. There will be a clear statement provided on the conditions under which participants should end their fast, for example, if they feel faint or unwell. After the study measurements are taken, a standardised post-fast meal or snack will be made available to participants. This will ensure that they can safely resume their regular dietary routines.

To verify adherence to the fasting protocol, participants will be provided with guidelines or a checklist. On the day of the study, researchers will verbally confirm with each

Rubio-Arias JÁ, *et al. BMJ Open Sp Ex Med* 2024; 9:e001797. doi: 10.1136/bmjsem-2023-001797

participant that they followed the fasting guidelines. To ensure the reliability and validity of our research findings, it is imperative to standardize the breakfast for all participants. By doing so, we eliminate potential variations in nutritional intake that could influence metabolic responses, thereby providing a consistent starting point for our study measurements. For breakfast, the total caloric intake will be set at 350 kcal. Carbohydrates constitute 60% of this, translating to 210 kcal or 53 grams. Proteins contribute 15%, which corresponds to 53 kcal or 13 grams. Fats, on the other hand, account for 25% of the meal's energy, amounting to 88 kcal or 10 grams.

a) Reproductive hormonal profile: to monitor the MC and to assess sexual function, serum concentration of reproductive system-related hormones will be analysed: 17β-estradiol, progesterone, prolactin, LH, FSH, TSH and testosterone [21], before each training session. The female hormonal profile will be measured basally in each phase of the menstrual cycle.

b) Inflammatory profile: although MS has been historically thought to be primarily a T-cell-driven disease, the role of B cells in the pathophysiology of MS has been increasingly recognised and characterised in recent years [22]. Therefore, IFN-γ, as a marker associated with the pathogenesis of the disease, IL-6 and TNF-α, as pro-inflammatory markers, and IL-10 and β1 (TGF-b1) as anti-inflammatory markers, will be assessed. All of these markers shall be assessed before and at 30 and 60 minutes immediately after the session [23]. For clinical analyses, the protocols described by Alvarenga-Filho et al. (2016) [24] will be followed. The inflammatory profile will be assessed pre-training, at 30 minutes, and 2 hours after the training session.

c) Cognitive function: brain-derived neurotrophic factor (BDNF) is a member of the neurotrophin family, and several studies have found associations between exercise, circulating levels of BDNF, hippocampal volume, and cognitive function in the general population [25] and neurodegenerative disorders [26]. For the analysis of BDNF, the protocol described by Briken et al. (2016) [27] will be followed, and BDNF will be assessed before, 30 minutes and 2 hours after the end of the training session. The inflammatory profile will be assessed pre-training, at 30 minutes, and 2 hours after the training session.

Neuromuscular Strength, Voluntary Activation, and Contractile Properties

a) Rate Force Development (RFD) and Maximal Voluntary Isometric Strength of the lower limbs (MVIC): following the recommendations by Maffiuletti et al. (2021) [28], the participants will be seated in a chair, with both legs flexed at a 90 degree angle and the ankle of those tested firmly strapped to a customised device with a load cell (MuscleLab Force Sensor, Ergotest AS, Langesund, Norway). Subsequently, participants will be

instructed to apply "as much force as possible, as fast as possible" on each trial. Participants will perform three maximal contractions lasting two seconds each, with a rest of three minutes between contractions. RFD and the time at which the value is observed will be analysed in the following ranges: 0-50 ms: RFD_{early}, 0-200 ms: RFD_{late} and peak slope: RFD_{peak}. The right leg will be tested first, and the repetition in which the highest peak value of each leg is observed will be analysed.

Ten minutes after the previous test and from the same position, in order to measure the maximum isometric force of each leg (MVIC), the participants will be verbally encouraged to apply "as much force as possible" during the course of 3 consecutive maximal contractions. Participants will perform three 5-second MVICs with 3 minutes of rest between contractions. The right leg will always be evaluated first, and the trial with the highest MVIC will be used for analysis.

To measure neural drive, the sEMG activity [29] of the vastus lateralis of the right leg will be recorded during the MVIC (Delsys Trigno, Delsys Inc., Boston, MA). The preparation of the skin will involve shaving, abrasion, and cleansing with alcohol. Following the SENIAM Guidelines [30], the upper electrode of each pair will be placed over the largest part of the vastus lateralis. Transparent paper will be used to map exact electrode placements for subsequent measurements. EMG activity will be analysed using the following time intervals: 0–50 ms (EMG 0 to 50), 0 to 200 ms (EMG 0 to 200), peak of EMG and EMG time-to-peak. The EMG peak during MVIC will represent maximal neural drive. [31]

In addition, two bipolar stimulating electrodes (10 × 15 cm) will be positioned and secured on the proximal and distal portions of the quadriceps of the right leg. Signal 6.0 software (CED, Cambridge, England) will control the electrical stimulation characteristics, which will be 100 Hz, 50 pulses, length 0.009 s, and interval 0.01 s. The stimulus intensity will be established at 40–50% of MVIC. Muscle contractile function and central activation ratio (CAR) will be assessed using the following protocol (Figure 4): a supramaximal twitch, a 100 Hz tetanic train (50 pulses), an MVIC with a superimposed 100 Hz train during steady maximal force, another potentiated 100-Hz train, and finally, a potentiated supramaximal twitch. This sequence will be repeated twice, providing 2 minutes of rest between the sequences. Twitch-to-tetanus ratio (Tw/Tet), peak MVIC, peak force obtained by the superimposed twitch and tetanus stimulation will be determined. The following formula will be used to calculate CAR:

$$CAR = \frac{MVIC}{MVIC + superimposed\ train} \times 100$$

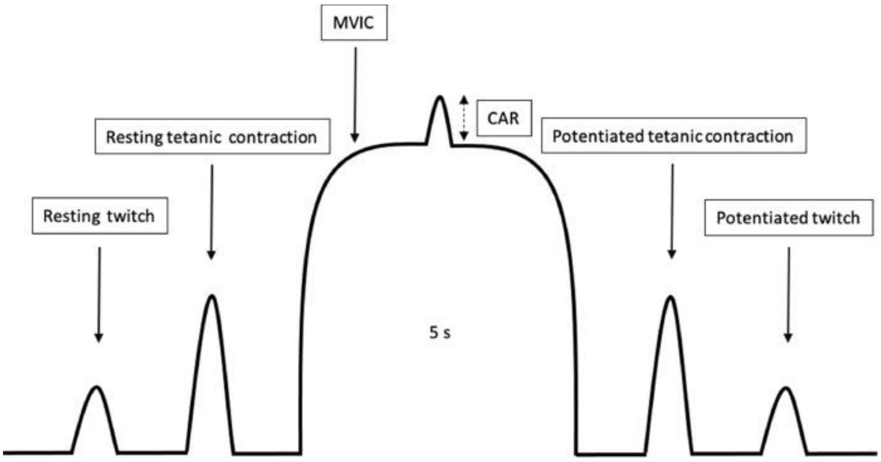

Figure 4. *Illustration of the Central Activation Ratio. CAR: Central Activation Ratio; MVIC: Maximal Voluntary Isometric Contraction.*

b) *Maximal upper limb strength (MULS)*: Participants will stand with their elbows fully extended and separated from the trunk. In this position, isometric handgrip strength will be measured for 5 seconds using the electronic hand dynamometer (K-Force Grip@, Kinvent, Monpellier, France[32]). Participants will perform three trials in each hand (right and left), and a rest interval of 30 seconds will be given between attempts. The highest value achieved will be recorded.

Functional assessment

a) Spasticity: The pendulum test will be used for spasticity assessment. Metrics including the relaxation index (RI), the count of oscillations, duration of oscillations, and the extent of the first swing excursion will be computed. Participants will sit with their torso reclined approximately 30° (to prevent biceps femoris stretching), and their legs will hang over the seat's edge. Participants will be barefoot and wearing shorts. Markers will be placed on the major trochanter of the femur, lateral epicondyle of the femur, and lateral malleolus of the fibula. A high-speed camera will record the pendulum test.

Participants will be instructed to close their eyes and ensure complete relaxation of their leg muscles throughout the testing. The examiner will hold the heel and move the leg from its resting position (approximately 90° knee flexion) to full extension (approximately 180° knee extension). Subsequently, the heel will be released to allow passive oscillation until the leg comes to rest. The examiner will continuously monitor the participant's relaxed leg during the test to verify trial validity. Rest periods of thirty seconds will be provided between trials. The analysis will be based on the mean of two valid trials.

Knee angles [33] will be determined using video recordings (Iphone 14-pro) for each pendulum test (Image J software, version 1.42; National Institutes of Health, Bethesda, MD, USA). The initial angle and the first inversion angle of the swinging limb will define the first swing excursion. The initial angle will be taken when the examiner releases the participant's heel. The count of swings will be ascertained from the number of sine wave peaks produced by the swinging limb following heel release. Each oscillation will be considered valid if it exhibits a shift of at least 3° towards extension. The oscillations' duration (in seconds) will also be determined from heel release until the end of the final oscillation defined by the aforementioned criterion. The RI will be calculated using the formula: RI = (initial angle - first angle) / (initial angle - rest angle). The rest angle will denote the knee joint's position after oscillatory motion concludes.

In addition, we will be using portable muscle diagnostic equipment (MyotonPRO, Myoton AS, Estonia) to assess muscle characteristics, including measurements of muscle frequency, muscle stiffness and muscle decline. The Myoton®PRO measures the deformation properties of damped natural oscillations that occur after a brief mechanical impact (15 ms) with a pre-compression of 0.18 N and an impulse of 0.40 N (total = 0.58 N) on the skin's surface. Anatomical locations will be selected to measure tone, stiffness and elasticity of the quadriceps and triceps surae muscles. The points defined by SENIAM for EMG [34] will be chosen for their reliability and reproducibility. First, a mark shall be made with a dermographic pen and then the marked point shall be measured. To ensure reproducibility, a permanent marker pen shall be used and the mark shall be checked by the patient himself every day to avoid erasure.

b) Gait speed: Gait speed will be measured using the 10-meter walk test (10-MWT), for which 2 photocells (Ergotest Technology AS, Langesund, Norway) will be placed at 5 and 10 meters to record the time. Participants will perform the test as fast as possible without running, completing it twice with a 2-minute rest in between. Participants will be consistently encouraged throughout the 10-MWT. The recorded time for the slowest walking trial (in seconds) will be utilized for analysis.

c) Balance: during the static balance measurements, participants will stand quietly on a portable force platform (Ergotest Technology AS, Langesund, Norway) while barefoot, maintaining a shoulder-width stance, and letting their arms hang at their sides. Each participant will perform two trials lasting 30 seconds each, with both eyes open, and another two trials of the same duration with their eyes closed. A rest period of two minutes will be allotted between trials. The trial yielding the most favourable balance outcomes for each test will be subjected to analysis. The outcome measures will be: the mean anterior/posterior displacement (MAPD; mm), mean medial/lateral displacement

(MMLD; mm), total sway displacement (TSD; mm), sway area (SA; mm2), mean total velocity (MTV; mm/s), phase plane portrait anterior/posterior (PPPAP; a.u.), medial/lateral (PPPML; a.u.) and anterior/posterior–medial/lateral (PPPAP-ML; a.u.), standard deviation of velocity anterior/posterior (SDVAP; mm/s) and medial/lateral (SDVML; mm/s), and standard deviation of amplitude anterior/posterior SDAAP (mm) and medial/lateral SDAML (mm). Balance will be tested before and after each session.
d) Sit-to-Stand Test (STS): Participants will be positioned in an upright manner on an adjustable chair (considering lower limb length; 90º knee flexion) with arms crossed over the chest. They will be instructed to rise to a standing position as swiftly as possible. Video recording with an Iphone ® 14 will be employed to determine the conclusion of the movement, when both the participant's trunk and knees are fully extended. This test will be conducted twice, and the most successful trial will be utilized for analysis [35].
e) Timed Up-and-Go Test (TUG): In a prompt manner, participants will transition from a seated to a standing position. They will then walk a distance of 3 meters forward, execute a turn, walk back, and resume the seated posture. This test will be repeated twice. A video recording will be used to ascertain the fastest time between the two trials, which will subsequently be used for analysis.

Pain

To measure Delayed-Onset Muscle Soreness (DOMS), a 10-point Likert scale will be employed to evaluate pain, where 1 = no pain and 10 = unbearable pain. Participants will assess their level of DOMS in nine different muscle areas: 1) posterior leg; 2) posterior thigh; 3) anterior thigh; 4) lower back and glutes; 5) abdominals; 6) upper back; 7) shoulders; 8) anterior arm; 9) posterior arm and total pain. A daily average score will be calculated by summing all pain scores and dividing it by the number of areas, to reflect overall DOMS. Additionally, an FPIX algometer (Wagner's instruments, USA) will be used to quantify pain sensitivity in specific muscle regions (same anatomical areas where spasticity is measured). The algometer will be calibrated according to the manufacturer's guidelines. Participants will be instructed to indicate their initial pain threshold. The rounded tip of the algometer will ensure an even application of pressure.

Rating of Perceived Exertion (RPE)

Participants will be instructed and familiarized with the use of the RPE scale during the familiarisation phase. RPE will be assessed before, during (after each of the proposed exercises within the session), and after the training session using the Borg 6-20 RPE [36,37].

1.3. Evaluations during intervention sessions (per)

Muscle oxygen saturation (SMO2)

SMO2 of the right and left lateral quadriceps will be measured during sessions using a near-infrared spectroscopy system, the Moxy 3-Sensor Bundle (Fortiori Design LLC, Hutchinson, MN, USA). The average SMO2 will be calculated throughout the entire training session on both legs simultaneously.

Lactate

A portable lactate analyser (Lactate Scout system, RedMed, Warsaw, Poland), which measures lactate concentration using the principle of enzymatic determination by photometric reflection, will be used. During the exercise sessions, capillary blood samples from the fingertip will be collected before warm-up (i.e. basal level) within the training session and after the end of cool down.

VO2max

Oxygen consumption (VO2) will be measured breath by breath throughout the session, and EPOC will be analysed for 20 minutes after the end of it, using a breath-by-breath gas analyser (VO2 Master Pro (VO2 Master Health Sensors Inc., Vernon, British Columbia, CA)). The gas analyser system will be calibrated before each test using the manufacturer's recommendations.

Heart rate variability

Autonomic nervous system adaptation. The fluctuation of the autonomic nervous system through the training session will be recorded. Blood pressure, oxygen saturation (SAT), heart rate (HR) and heart rate variability (HRV) will be recorded. The systolic (SBP) and diastolic blood (DBP) pressure will be obtained by a single indirect measurement performed by an automatic oscillometric blood pressure measuring device (OMRON, Kyoto, Japan) positioned on the left arm of the patient. Oxygen saturation will be measured using a portable finger oximeter (9500, Onix-Indumeda, Nonin Fernbrook Lane North Plymouth, USA) positioned on the third finger of the participant. Resting, reactivity and recovery HR and HRV will be recorded using a Polar H10 heart rate sensor (Kempele, Finland). HRV time-domain and frequency-domain will be recorded and logarithm-transformed as described in the quality of sleep section. For that purpose, the participant will be in a supine position for 10 minutes before and after the training session. Resting HR and HRV will correspond with the 10 minutes of rest before the training session [38], reactivity HR and HRV will comprise the period of exercise, removing the first and last 5 minutes, and recovery HR and HRV will correspond to the 10 minutes of rest after the training session, which will be subdivided into two phases: the first ($HR_{rec}1$ and $HRV_{rec}1$) and the last ($HR_{rec}2$ and $HRV_{rec}2$) 5 minutes of recovery.

1.4. Residual effect of exercise training sessions

Quality of sleep will be measured during the 3 nights following the intervention.

a) Subjective Sleep Quality Questionnaire: Subjective sleep quality will be measured using the Karolinska Sleep Diary questionnaire [39]. The questionnaire includes the following items: a) sleep quality (very poor [1] – very good [5]), b) sleep tranquillity (very restless [1] – very calm [5]), c) ease of falling asleep (very difficult [1] – very easy [5]), d) awakenings (awakened much too early [1] – did not wake up early [3]), e) ease of awakening (very difficult [1] – very easy [5]), f) feeling of restfulness (did not rest at all [1] – completely rested [3]), and g) sleep sufficiency (no, definitely too little [1] – yes, definitely enough [5]).

b) Sleep Quality Measured by Actigraphy: Actigraphy-based sleep quality will be assessed using the Actiwatch wGT3X-BT activity monitoring system (Cambridge Neurotechnology, Cambridge, UK). This device employs a piezoelectric accelerometer to measure activity. Participants will wear the Actiwatch on their non-dominant wrist. The lower threshold of actigraphic sensitivity will be set at 80 counts/epoch. The Actiwatch sleep analysis software will be utilized. Data analysis will begin at the start of the nocturnal rest (bedtime) and conclude at the beginning of daytime activity (wake time). Sleep efficiency (%), percentage of time spent asleep, time in bed (minutes), actual sleep time (minutes), actual wake time (minutes), number of awakenings, and average duration of each awakening (minutes) will be measured.

A minimum of 2 nights is required to evaluate sleep percentage, at least 5 nights for sleep efficiency, and 7 nights for total sleep time using Actigraphy. Self-reported questionnaires require a minimum of 6 nights [39].

c) HRV: HRV measurements will be taken using a Polar H10 heart rate sensor (Kempele, Finland) to record heartbeats during sleep. Analysis of HRV variables will be conducted using Kubios HRV software (version 3.0). This software will also be used to apply threshold filters (very low, low, or medium) to eliminate artefacts where necessary. Poincaré plot, time-domain, and frequency-domain analyses will be performed. The analysed time-domain variables include [40]: mean heart rate (HR), mean R-R interval (RR; ms), standard deviation of consecutive R-R intervals (SDNN), square root of the mean squared differences of successive R-R intervals (RMSSD; ms), and the proportion of consecutive intervals differing by more than 50 ms (pNN50). Fast Fourier Transformation (FFT) will be employed to calculate frequency-domain spectral components. High-frequency (HF; 0.15-1.0 Hz) and low-frequency (LF; 0.04-0.15 Hz) power components will be computed as integrals of their respective power spectral density curves. These

variables will be expressed as natural logarithm-transformed values (HFln and LFln). Lastly, Poincaré plot variables, standard deviation of instantaneous beat-to-beat variability (SD1), and standard deviation of continuous long-term R-R interval variability (SD2) will be calculated. Stress score (SS) will be analysed using the equation 1000x1/SD2, and the sympathetic/parasympathetic ratio (S/PS) will be calculated using SS/SD1.

References:

1. Stewart AD, Marfell-Jones M, Olds T, Hans De Ridder J. *International Standards for Anthropometric Assessment*.; 2011.
2. Duda K, Majerczak J, Nieckarz Z, Heymsfield SB, Zoladz JA. Human Body Composition and Muscle Mass. *Muscle and Exercise Physiology*. Published online January 1, 2019:3-26. doi:10.1016/B978-0-12-814593-7.00001-3
3. Woolcott OO, Bergman RN. Relative fat mass (RFM) as a new estimator of whole-body fat percentage – A cross-sectional study in American adult individuals. *Sci Rep*. 2018;8(1):10980. doi:10.1038/s41598-018-29362-1
4. Yuksel H, Balaban M, Tan OO, Mungan S. Sarcopenia in patients with multiple sclerosis. *Mult Scler Relat Disord*. 1800;58:103471.
5. Cruz-Jentoft AJ, Bahat G, Bauer J, et al. Sarcopenia: revised European consensus on definition and diagnosis. *Age Ageing*. 2019;48(4):601. doi:10.1093/AGEING/AFZ046
6. Schröder H, Fitó M, Estruch R, et al. A Short Screener Is Valid for Assessing Mediterranean Diet Adherence among Older Spanish Men and Women. *J Nutr*. 2011;141(6):1140-1145. doi:10.3945/jn.110.135566
7. Álvarez-Álvarez I, Martínez-González MÁ, Sánchez-Tainta A, et al. Dieta mediterránea hipocalórica y factores de riesgo cardiovascular: análisis transversal de PREDIMED-Plus. *Rev Esp Cardiol*. 2019;72(11):925-934. doi:10.1016/j.recesp.2018.08.007
8. Melguizo-Ibáñez E, González-Valero G, Badicu G, et al. Mediterranean diet adherence on self-concept and anxiety as a function of weekly physical activity: an explanatory model in higher education. *Front Nutr*. 2023;10. doi:10.3389/fnut.2023.1215359
9. García-Conesa MT, Philippou E, Pafilas C, et al. Exploring the Validity of the 14-Item Mediterranean Diet Adherence Screener (MEDAS): A Cross-National Study in Seven European Countries around the Mediterranean Region. *Nutrients*. 2020;12(10):2960. doi:10.3390/nu12102960
10. Fortune J, Norris M, Stennett AM, et al. Validity of the international physical activity questionnaire (IPAQ-s) as a measure of physical activity in people with multiple sclerosis. *Physiotherapy*. 2019;105:e19. doi:10.1016/J.PHYSIO.2018.11.260
11. Roman-Viñas B, Serra-Majem L, Hagströmer M, Ribas-Barba L, Sjöström M, Segura-Cardona R. International Physical Activity Questionnaire: Reliability and validity in a

Spanish population. *https://doi.org/101080/17461390903426667*. 2010;10(5):297-304. doi:10.1080/17461390903426667

12. Gijbels D, Eijnde B, Feys P. Comparison of the 2- and 6-minute walk test in multiple sclerosis. *Multiple Sclerosis Journal*. 2011;17(10):1269-1272. doi:10.1177/1352458511408475

13. Grandmontagne AG, Azúa SR, Liberal I. Propiedades psicométricas de un nuevo cuestionario para la medida del autoconcepto físico. *Rev Psicol Deporte*. 2004;13(2):195-213.

14. Sullivan MJL, Bishop SR, Pivik J. The Pain Catastrophizing Scale: Development and Validation. *Psychol Assess*. Published online 1995. doi:10.1037/1040-3590.7.4.524

15. Rooney S, McFadyen DA, Wood DL, Moffat DF, Paul PL. Minimally important difference of the fatigue severity scale and modified fatigue impact scale in people with multiple sclerosis. *Mult Scler Relat Disord*. 2019;35:158-163. doi:10.1016/j.msard.2019.07.028

16. Spielberger CD, Reheiser EC. Measuring anxiety, anger, depression, and curiosity as emotional states and personality traits with the STAI, STAXI and STPI. In: *Comprehensive Handbook of Psychological Assessment Vol. 2 Personality Assessment*. ; 2004.

17. Moreira DG, Costello JT, Brito CJ, et al. Thermographic imaging in sports and exercise medicine: A Delphi study and consensus statement on the measurement of human skin temperature. *J Therm Biol*. 2017;69:155-162. doi:10.1016/j.jtherbio.2017.07.006

18. Ammer K. The Glamorgan Protocol for recording and evaluation of thermal images of the human body. *Thermology International*. 2008;18:125-129.

19. Fernández-Cuevas I, Bouzas Marins JC, Arnáiz Lastras J, et al. Classification of factors influencing the use of infrared thermography in humans: A review. *Infrared Phys Technol*. 2015;71:28-55. doi:10.1016/j.infrared.2015.02.007

20. Marins J, Gomes Moreira D, Cano S, et al. Time required to stabilize thermographic images at rest. *Infrared Phys Technol*. Published online October 2014. doi:10.1016/j.infrared.2014.02.008

21. Peinado AB, Alfaro-Magallanes VM, Romero-Parra N, et al. Methodological Approach of the Iron and Muscular Damage: Female Metabolism and Menstrual Cycle during Exercise Project (IronFEMME Study). *International Journal of Environmental Research and Public Health 2021, Vol 18, Page 735*. 2021;18(2):735. doi:10.3390/IJERPH18020735

22. Goldman MD, Ward M, Goldman MD. Epidemiology and Pathophysiology of Multiple Sclerosis. *Continuum (Minneap Minn)*. 2022;28(4):988-1005. doi:10.1212/CON.0000000000001136

23. Castellano V, Patel DI, White LJ. Cytokine responses to acute and chronic exercise in multiple sclerosis. *J Appl Physiol (1985)*. 2008;104(6):1697-1702. doi:10.1152/japplphysiol.00954.2007

24. Alvarenga-Filho H, Sacramento PM, Ferreira TB, et al. Combined exercise training reduces fatigue and modulates the cytokine profile of T-cells from multiple sclerosis

patients in response to neuromediators. *J Neuroimmunol*. 2016;293:91-99. doi:10.1016/J.JNEUROIM.2016.02.014

25. Smith PJ, Blumenthal JA, Hoffman BM, et al. Aerobic Exercise and Neurocognitive Performance: a Meta-Analytic Review of Randomized Controlled Trials. *Psychosom Med*. 2010;72(3):239. doi:10.1097/PSY.0B013E3181D14633

26. Ruiz-González D, Hernández-Martínez A, Valenzuela PL, Morales JS, Soriano-Maldonado A. Effects of physical exercise on plasma brain-derived neurotrophic factor in neurodegenerative disorders: A systematic review and meta-analysis of randomized controlled trials. *Neurosci Biobehav Rev*. 2021;128:394-405. doi:10.1016/J.NEUBIOREV.2021.05.025

27. Briken S, Rosenkranz SC, Keminer O, et al. Effects of exercise on Irisin, BDNF and IL-6 serum levels in patients with progressive multiple sclerosis. *J Neuroimmunol*. 2016;299:53-58. doi:10.1016/J.JNEUROIM.2016.08.007

28. Maffiuletti NA, Aagaard P, Blazevich AJ, Folland J, Tillin N, Duchateau J. Rate of force development: physiological and methodological considerations. *Eur J Appl Physiol*. Published online 2016. doi:10.1007/s00421-016-3346-6

29. Kent-Braun JA. Central and peripheral contributions to muscle fatigue in humans during sustained maximal effort. *Eur J Appl Physiol Occup Physiol*. 1999;80(1):57-63. doi:10.1007/s004210050558

30. Hermens HJ, Rau G, Disselhorst-Klug C, Freriks B. Surface Electromyography Application Areas and Parameters (SENIAM 3). *Standards for surface electromyography: the European project (SENIAM)*. 1998;(May):108-112.

31. Aagaard P, Simonsen EB, Andersen JL, Magnusson P, Dyhre-Poulsen P. Increased rate of force development and neural drive of human skeletal muscle following resistance training. *J Appl Physiol*. Published online 2002. doi:10.1152/japplphysiol.00283.2002

32. Nikodelis T, Savvoulidis S, Athanasakis P, Chalitsios C, Loizidis T. Comparative Study of Validity and Reliability of Two Handgrip Dynamometers: K-Force Grip and Jamar. *Biomechanics*. 2021;1(1):73-82. doi:10.3390/biomechanics1010006

33. Fowler EG, Nwigwe AI, Ho TW. Sensitivity of the pendulum test for assessing spasticity in persons with cerebral palsy. *Dev Med Child Neurol*. Published online 2000. doi:10.1017/S0012162200000323

34. Hermens HJ, Freriks B, Disselhorst-Klug C, Rau G. Development of recommendations for SEMG sensors and sensor placement procedures. *Journal of Electromyography and Kinesiology*. 2000;10(5):361-374. doi:10.1016/S1050-6411(00)00027-4

35. Ruiz-Cárdenas JD, Rodriguez Juan JJ. Sit To Stand. Published online 2017.

36. Borg G. Psychophysical scaling with applications in physical work and the perception of exertion. *Scand J Work Environ Health*. 1990;16(SUPPL. 1):55-58. doi:10.5271/SJWEH.1815

37. Cleland BT, Ingraham BA, Pitluck MC, Woo D, Ng A V. Reliability and Validity of Ratings of Perceived Exertion in Persons With Multiple Sclerosis. *Arch Phys Med Rehabil*. 2016;97(6):974-982. doi:10.1016/j.apmr.2016.01.013

38. Raimundo RD, Zangirolami-Raimundo J, Leone C, et al. The Use of Cardiac Autonomic Responses to Aerobic Exercise in Elderly Stroke Patients: Functional Rehabilitation as a Public Health Policy. *Int J Environ Res Public Health*. 2021;18(21):11460. doi:10.3390/ijerph182111460

39. Åkerstedt T, Hume K, Minors D, Waterhouse J. The meaning of good sleep: a longitudinal study of polysomnography and subjective sleep quality. *J Sleep Res*. 1994;3(3):152-158. doi:10.1111/J.1365-2869.1994.TB00122.X

40. Laborde S, Mosley E, Thayer JF. Heart Rate Variability and Cardiac Vagal Tone in Psychophysiological Research – Recommendations for Experiment Planning, Data Analysis, and Data Reporting. *Front Psychol*. 2017;08. doi:10.3389/fpsyg.2017.00213
